# Protocol for middle cerebral artery occlusion by an intraluminal suture method

**DOI:** 10.4103/0976-500X.77113

**Published:** 2011

**Authors:** M. Rupadevi, S. Parasuraman, R. Raveendran

**Affiliations:** *Department of Pharmacology, Jawaharlal Institute of Postgraduate Medical Education and Research, Pondicherry – 605 006, India*

## INTRODUCTION

Generally, the experimental animal models serve as indispensable tools to predict the value and the effect of therapeutic approaches in human subjects. A number of procedures have been followed to develop experimental models of stroke in many species. The rodents (rats and mice) are involved in the majority of neurological experiments, since they are cost effective, free of ethical burdens compared to larger animals, and especially, their cerebrovascular anatomy and physiology presents resemblance to humans. Hemorrhagic, global, and focal ischemia are the general approaches to establish the animal models of stroke. Among these approaches, the focus on the focal ischemic models has increased, with preference for middle cerebral artery occlusion (MCAo). In the MCAo procedure, there are two major variants, i.e., transient and permanent. Initially, the direct middle cerebral artery (MCA) ligation by a subtemporal approach was considered as the standard method of producing a permanent proximal MCAo but MCA occlusion by craniectomy may cause damage due to brain retraction and vessel manipulation. Apart from this disadvantage, reperfusion may not be attained easily and this technique can disturb the intracranial environment due to its surgically invasive nature. Hence, MCAo using intraluminal suture has gained increasing attention in the stroke research. MCAo by the intraluminal suture method in rodents is a widely accepted and well-standardized animal model for cerebral ischemia and reperfusion injury. Kozuimi *et al*.[1] introduced this model which mimics the human ischemic stroke and produces infarction in the MCA territory which involves both frontopareital cortex and lateral caudoputamen. MCAo is performed via either external carotid artery or the common carotid artery which eliminated the requirement of craniectomy and all the problems associated with an open skull procedure. The major advantage of this technique is that reperfusion can be achieved easily which helps to alter the duration of ischemia in a controlled manner. This article describes the methodology and evaluation of the MCAo model in rodents. The approval from the relevant committees (Institutional animal ethics committee) is required for all the experimental procedures.

## PREOPERATIVE CARE

- Healthy adult male rats weighing 200–250 g or mice weighing 25–30 g are selected for the surgery. Since estrogen is a neuroprotectant which may affect the intensity of infarction,[2] males are preferable.
- All the animals are fasted overnight before the experiment.

## PREPARATION OF THE MONOFILAMENT FOR MCAo

Requirements include 6-0 (mice) or 4-0 (rats) nylon monofilament (Ethicon, Johnson and Johnson), a candle, forceps, and poly-L-lysine (0.1% w/v; Sigma).

- The monofilament is cut into 2 cm (for mice) or 3 cm (for rats) pieces and the tip of the monofilament is heated for a brief period or till the tip is swollen to form a bulb shape or blunt end (all the tip ends are uniformly blunted to occlude the MCA).
- The monofilaments are immersed in poly-L-lysine for 30 min and dried in an incubator at 37 ± 2°C for overnight. The filaments are stored for surgical purpose.
- Poly-L-lysine is used as an adhesive subbing solution and its polycationic nature helps to facilitate a strong interaction with the anionic sites of the artery which produces a complete occlusion of the MCA.
- The blunt end of the filament occludes the MCA properly which helps to produce ischemia in the MCA territory.
- It is recommended to standardize the optimal tip diameter and inserted length of the suture for the animal used. To get 100% successful stroke, silicone-coated filaments are preferable, and are commercially available.

## PROCEDURE FOR MCAo

All the surgical instruments and materials should be autoclaved and the surgical procedure should be performed under sterile conditions.

Requirements include ketamine, xylazine, normal saline, surgical gloves, adhesive tape, curved scissors, surgical blade, curved forceps, microscissors, scissors, microvascular clips, cotton thread, suture, 1-ml syringe, sterilized cotton, 2% lidocaine HCl (Xylocaine), dissection microscope (for mice), and needle (size 16 for rats).

- Animals are anesthetized with ketamine (80 mg/kg) and xylazine (10 mg/kg) intraperitoneally or induced and maintained with 3% and 1.5% isoflurane, respectively, along with 80% oxygen using a vaporizer (it has been reported that the inhalation of anesthesia is effective compared to other methods due to the better control of physiological parameters and lower mortality during 7 days postischemia).[3]
- After turning the animal to the supine position, it is fixed to the surgical table using an adhesive tape.
- A midline neck incision is made and the soft tissues over the trachea are retracted gently.
- The common carotid artery (CCA) either left or right is carefully isolated from the vagus nerve and ligated temporarily using a cotton thread.
- Generally, the CCA is bifurcated into the external carotid artery (ECA) and internal carotid artery (ICA) which flows toward the head region, and again bifurcated into the MCA and pterigopalatine artery.
- The first bifurcation of CCA is identified, and then, the soft tissues around the ECA and ICA are gently cleared without harming the artery.
- Then, two closely spaced permanent knots are placed at the distal part of the ECA to prevent the backflow of blood and the ECA is cut between the knots.
- The tied section attached proximal to the CCA junction can be straightened to allow the filament to enter the ICA, and then, the second bifurcation is cleared to obtain a good view of the MCA.
- The microvascular clip is placed in the ICA temporarily proximal to the CCA junction and the tied section of ECA is incised using the microscissors to insert the monofilament. Once the tip of the monofilament reaches the CCA junction, a knot is placed below the arteriotomy in the ECA, and then, the microvascular clip which is placed in the ICA is removed permanently to allow filament insertion.
- The ECA stump is straightened and the filament is advanced carefully up to 11 mm, for mice, or 17–20 mm,[4] for rats, into the MCA from the CCA junction.
- Once filament insertion into the MCA is confirmed, the microvascular clip is removed permanently from the CCA and animal’s body temperature is maintained at 37 ± 2°C using a heating blanket during occlusion.
- After the specific occlusion period, again a clip is placed in the ICA and the knot placed in the ECA stump below the arteriotomy is loosened. The intensity of the infarction greatly depends on the MCA occlusion period. The minimum 60–90 min of the occlusion period is required to obtain a reproducible infarct volume,[5] and therefore, the most common occlusion periods of the MCA are 60, 90, and 120 min for rats.[6]
- The filament is then withdrawn carefully until the tip is near the arteriotomy.
- After the removal of the filament, the knot is tightened in the ECA. After the confirmation of anterograde blood flow restoration (reperfusion), the midline neck incision is sewed using surgical suture.
- To relieve pain and discomfort in the postoperative period, topical Lidocaine gel is applied on the wound and the animal receives 0.5 ml saline intraperitoneally as volume replenishment after the surgery.
- At the end point of the study, the animals are sacrificed and the histological analysis is carried out to confirm infarction. Generally, brain infarction can be observed after 23 h of reperfusion or surgery.

## PHYSIOLOGICAL PARAMETERS

- Mean arterial blood pressure, heart rate, and blood gases can be measured to monitor the complications during surgery.
- The measurement of the cerebral blood flow with laser Doppler flowmetry during surgery ensures the proper occlusion of the MCA.

## PROCEDURE FOR SHAM SURGERY

- For sham surgery, all the arteries are exposed for the surgical period but the filament is not inserted into the MCA. The surgical period and the anesthesia volume should be same as that for the test animal.

## POSTOPERATIVE CARE

- Ensure that there is no subarachnoid hemorrhage, the lesion is induced and no other complications are noted.
- The animals which are having problems during the induction of MCAo, such as excessive bleeding, prolonged operation time, and thread displaced into the pterygopalatine artery, are excluded.
- The animal must be monitored carefully after surgery for signs of discomfort, and caged individually.

## NEUROLOGICAL DEFICITS AFTER STROKE INDUCTION

- The acute neurological deficit of the animal is evaluated by placing the rat on the floor (normal walk = 0, inability to walk straight = 1, circling toward the paretic side = 2, fall down to the paretic side=3) and motor test (flexion of the forelimb and hind limb) after recovered from anesthesia.[7]

## EVALUATION OF BRAIN INFARCTION BY HISTOLOGICAL ANALYSIS

Requirements include chloroform, surgical blade/brain matrix, 2,3,5-triphenyltetrazolium chloride (TTC), normal saline, 100-ml beaker, rat or mouse brain 10% formalin.

## PREPARATION OF THE REAGENT

- 0.9% NaCl
- 0.5% TTC (for 50 ml of 0.9% NaCl, mix 500 mg of TTC).

The solution should be prepared freshly.

## PRINCIPLE

TTC is an oxidation–reduction indicator which is used for early histochemical diagnosis of infarction and is one of the most frequently used staining method for the reliable macroscopic identification of infarction.[8,9] The staining action of TTC is based on the oxidation of TTC by intact mitochondrial dehydrogenase, which oxidizes the tetrazolium salts into formazan, the carmine red product. Due to the absence of active dehydrogenase in the infracted or necrotic tissue, it remains unstained. Therefore, the infracted tissue can be visually identified.

## PROCEDURE FOR TTC STAINING

- After 24 h of reperfusion, the animal is euthanized using the overdose of urethane followed by cervical dislocation and decapitated to remove the brain carefully.
- The brain is frozen at –80°C for 15 min and sliced into 1-mm (for mice) or 2-mm (for rats) coronal sections.
- The brain slices are immersed in the TTC solution for 15–20 min at 37°C.
- The ischemic damage to coronal brain sections is qualified by the absence of staining and, for fixation, the brain slices are then transferred into a 10% v/v formaldehyde solution after washing with normal saline.
- Images of brain sections are captured using a digital camera with good resolution.

## DISCUSSION

Experimental ischemic stroke models render a better understanding of the mechanisms of an ischemic brain injury. Despite tremendous efforts that have been made in the last two decades, the recombinant tissue-type plasminogen activator is the only approved therapy for acute ischemic stroke within a 3-h time window, and there are many failures of neuroprotective drug clinical studies. Though many reasons can be interpreted for these failures, the drawbacks of preclinical studies such as false-positive results, inflated effect size, and marginal reproducibility have to be considered. MCAo by the intraluminal suture method is a better choice for experimental models of stroke since it may produce a more reproducible alternative for inducing stroke. Moreover, it yields highly consistent results which minimize misleading interpretations, and hence, it is considered as a reliable stroke model to test a variety of neuroprotective drugs.
